# An Apicoplast Localized Ubiquitylation System Is Required for the Import of Nuclear-encoded Plastid Proteins

**DOI:** 10.1371/journal.ppat.1003426

**Published:** 2013-06-13

**Authors:** Swati Agrawal, Duk-Won D. Chung, Nadia Ponts, Giel G. van Dooren, Jacques Prudhomme, Carrie F. Brooks, Elisadra M. Rodrigues, John C. Tan, Michael T. Ferdig, Boris Striepen, Karine G. Le Roch

**Affiliations:** 1 Department of Cellular Biology, University of Georgia, Athens, Georgia, United States of America; 2 Department of Cell Biology and Neuroscience, University of California, Riverside, California, United States of America; 3 Center for Tropical and Emerging Global Diseases, University of Georgia, Athens, Georgia, United States of America; 4 Research School of Biology, Australian National University, Canberra, Australia; 5 Eck Institute of Global Health, University of Notre Dame, South Bend, Indiana, United States of America; University of Geneva, Switzerland

## Abstract

Apicomplexan parasites are responsible for numerous important human diseases including toxoplasmosis, cryptosporidiosis, and most importantly malaria. There is a constant need for new antimalarials, and one of most keenly pursued drug targets is an ancient algal endosymbiont, the apicoplast. The apicoplast is essential for parasite survival, and several aspects of its metabolism and maintenance have been validated as targets of anti-parasitic drug treatment. Most apicoplast proteins are nuclear encoded and have to be imported into the organelle. Recently, a protein translocon typically required for endoplasmic reticulum associated protein degradation (ERAD) has been proposed to act in apicoplast protein import. Here, we show ubiquitylation to be a conserved and essential component of this process. We identify apicoplast localized ubiquitin activating, conjugating and ligating enzymes in *Toxoplasma gondii* and *Plasmodium falciparum* and observe biochemical activity by *in vitro* reconstitution. Using conditional gene ablation and complementation analysis we link this activity to apicoplast protein import and parasite survival. Our studies suggest ubiquitylation to be a mechanistic requirement of apicoplast protein import independent to the proteasomal degradation pathway.

## Author Summary

The apicoplast is an essential parasite organelle derived from an algal endosymbiont. Most apicoplast proteins are nuclear encoded and post-translationally imported. Part of this journey utilizes the endoplasmic reticulum associated degradation or ERAD system of the algal endosymbiont. Typically, the ERAD system is ubiquitylation-dependent and acts in the retrotranslocation across the ER membrane and proteasomal destruction of misfolded secretory proteins. In the apicoplast, this system has been retooled into a protein importer. The apicoplast ERAD system is broadly conserved between most apicomplexans and surprisingly retains the ubiquitylation machine typically associated with destruction. This study brings together biochemical studies in *Plasmodium* and genetic studies in *Toxoplasma*. Together they provide significant mechanistic insight into the process of protein import into the apicoplast. We provide evidence that ubiquitylation may be a mechanistic requirement for import and demonstrate it to be essential to the parasite, thus providing new opportunities for drug development.

## Introduction

Apicomplexans are eukaryotic pathogens and responsible for important human and animal diseases including malaria and toxoplasmosis. The Apicomplexa evolved from single-celled photosynthetic algae, and their adaptation to animal parasitism likely predates the emergence of animals from water to land. The presence of a plastid, the apicoplast, is the most important remnant of this evolutionary past [1], [2]. While no longer photosynthetic, the organelle synthesizes isoprenoids and fatty acids [3]. The apicoplast is essential for parasite survival, and its metabolism, biogenesis and maintenance are important targets for anti-parasitic drug treatment. The apicoplast was derived by secondary endosymbiosis, where a unicellular red alga was incorporated into a heterotrophic protist. As a consequence of this secondary endosymbiosis the apicoplast is surrounded by four membranes. The organelle carries a genome, yet most of its proteins are nuclear-encoded and imported into the organelle after translation. Targeting depends on a bipartite leader peptide, the first section of which mediates co-translational import into the endoplasmic reticulum, and the second part mediates delivery to the apicoplast, likely through fusion of endosomal vesicles with the outermost membrane of the organelle [4]. Three translocons breaching successive membranes have been proposed to act in the further transport of proteins into the stroma of the apicoplast [5]. The two inner membranes of the apicoplast are homologous to the membranes of the primary chloroplast and protein transport depends on systems derived from the chloroplast TIC and TOC machinery [6], [7], [8], [9]. Insight into the third translocon emerged first in cryptomonads, an algal group that like Apicomplexa harbors a secondary plastid. The secondary plastids of cryptomonads retained a remnant of the algal nucleus, the nucleomorph. Analysis of the gene content of the nucleomorph led to the discovery of plastid proteins that resembled components of the endoplasmic reticulum associated degradation (ERAD) machinery [10]. ERAD is a quality control mechanism that retro-translocates misfolded secretory proteins across the ER membrane [11]. Sommer and colleagues proposed that this mechanism has been adapted for protein import in secondary plastids [10]. There is now significant support for this hypothesis. Homologs of ERAD proteins have been identified and localized to plastids in various algal and apicomplexan species including a core of the membrane protein Der1, the AAA ATPase Cdc48 and its cofactor Ufd1 [10], [12], [13], [14], [15]. Recombinant plastid proteins can complement yeast ERAD mutants [14]. Importantly, genetic ablation of the ERAD component Der1_Ap_ in *T. gondii* blocks apicoplast protein import, producing a phenotype that closely resembles ablation of the apicoplast TIC component Tic20 [6], [15].

During classical ERAD, protein translocation coincides with ubiquitylation, a process that typically employs a cascade of three enzymes: ubiquitin-activating enzyme (E1), ubiquitin-conjugating enzyme (E2), and ubiquitin ligase (E3) [16], [17]. Consuming ATP, the E1 enzyme adenylates ubiquitin at the C-terminus, creating a mixed anhydride. The sulfhydryl group of the E1 active-site cysteine then attacks the anhydride, which results in the formation of a high-energy thio-ester linking ubiquitin to E1. Ubiquitin is then passed to the active-site cysteine of the E2 enzyme. Lastly, with the aid of an E3 ligase, ubiquitin is transferred from E2 and covalently attached to the ε-amino group of a lysine in the target protein. Although clearly important in mediating ERAD, the role of ubiquitylation in protein import into secondary plastids is unclear. Interestingly, some ERAD-like ubiquitylation factors are observed in the plastids of cryptomonads, diatoms, and Apicomplexa [12], [18], [19].

While protein degradation is the key function of classical ERAD this could seem counterintuitive in the context of apicoplast protein import. However ubiquitin's functions are not limited to proteasomal degradation and extend to a variety of cellular protein trafficking systems [20]. Furthermore, ubiquitylation may be a critical mechanistic requirement of protein transport *via* the ERAD translocon [11], [21]. Some authors now view the ERAD associated E3 ligase Hrd1 as a favored candidate for the actual protein-conducting pore [22].

In this study, we elucidate the function of ubiquitylation in the apicoplast. We identify and localize a comprehensive set of ubiquitylating components in the apicomplexan parasites *P. falciparum* and *T. gondii*. Using recombinant apicoplast enzymes from *P. falciparum* we reconstitute ubiquitylation *in vitro* using a variety of heterologous and homologous cofactors. By genetic analysis in *T. gondii* we demonstrate that loss of the apicoplast-localized ubiquitin-conjugating enzyme leads to loss of apicoplast protein import and parasite demise. Importantly complementation of this mutant depends on an active site cysteine required for enzymatic activity. Taken together our experiments suggest an essential mechanistic role for the ERAD-like ubiquitylation machinery in apicoplast protein import.

## Results

### Ubiquitylation factors localize to the apicoplast in *Toxoplasma* and *Plasmodium*

**Figure 1:**
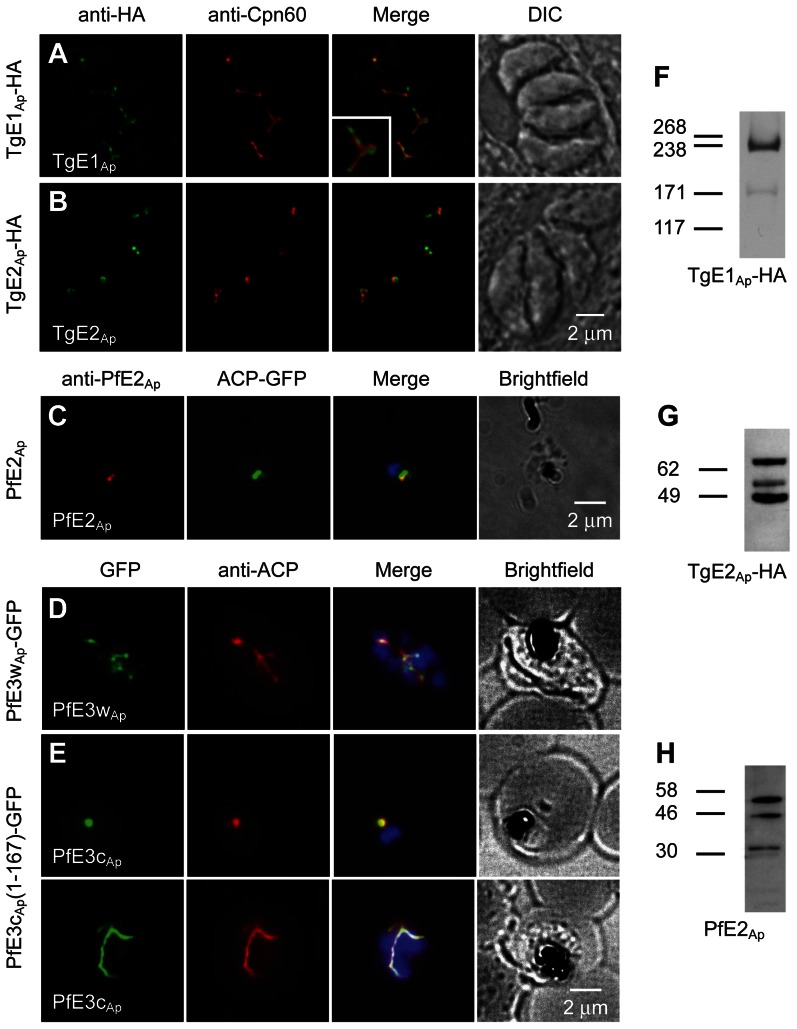
Ubiquitylation proteins localize to the apicoplast in *Plasmodium falciparum* and *Toxoplasma gondii*. (**A–E**) Immunofluorescence assays detecting the respective ubiquitylation factor indicated (white lettering) in the left-most panel. Proteins were detected by tagging with an HA or GFP epitope in the genomic locus (**A**, **D**), by ectopic fusion with full (**B**) or partial coding sequence (**E**), or by using an antibody raised again recombinant protein (**C**). Staining for the apicoplast markers Cpn60 and ACP is shown in the second lane. Merged images also show DAPI staining for *P. falciparum*. Insert in (**B**) shows a 200% enlargement. Western blots of *T. gondii* (**F**, **G**, transgene as indicated) or *P. falciparum* (**H**) protein samples reacted with anti-HA and anti-PfE2_Ap_ antibodies. Predicted sizes of initial translation product of TgE2_Ap_, PfE2_Ap_ and TgE1_Ap_ are 70.4 kDa, 32.8 kDa and 315.9 kDa respectively.

Using a combination of computational approaches we identified a comprehensive set of proteins that may act as apicoplast ubiquitylation system (see Materials and Methods). The results of these analyses (summarized in Table S1 in Text S1) identified apicoplast candidates for E1, E2 and E3 enzymes in both *P. falciparum* and *T. gondii*. We next determined whether these candidates are indeed targeted to the apicoplast. We targeted the locus of *T. gondii* TgE1_Ap_ by single homologous integration and placed a haemagglutinin (HA) epitope tag at the C-terminus of the protein. Stable transgenic clones show apicoplast staining when labeled with an anti-HA antibody by immunofluorescence (Fig. 1A, the *P. falciparum* homolog E1 is also localized to the apicoplast [12]). Our attempts to localize the candidates for apicoplast E2 by tagging the respective genes directly in the locus did not produce viable transgenics in either *T. gondii or P. falciparum*. Epitope fusion close to the C-terminal active domain may interfere with function and prevent replacement of the native gene. However, the coding sequence of TgE2_Ap_ could be fused to an epitope tag in the context of an ectopic expression plasmid (maintaining the native locus). Parasites expressing this construct show apicoplast labeling indistinguishable from that observed for E1 when probed with an epitope specific antibody. To localize the *Plasmodium* homolog (and to aid subsequent biochemical analysis) we also expressed a portion of Mal13P1.227 fused to an affinity tag in *E. coli* and used the purified recombinant protein to raise a specific antiserum. Immunofluorescence assays on *P. falciparum* parasites with this serum produced labeling that coincides with labeling for the apicoplast marker ACP (Fig. 1 C).

Two putative apicoplast E3 ubiquitin ligases were identified in *Plasmodium*, PfE3c_Ap_ (PFC0740c - PF3D7_0316900) and PfE3w_Ap_ (PFC0510w - PF3D7_0312100), and two in *Toxoplasma* (TGME49_226740 and TGME49_304460). We attempted to tag the proteins by placing different epitopes at the C-terminus through homologous gene targeting but were not successful. In case of PfE3c_Ap_ transgenics that showed initial locus targeting were quickly lost upon selection (Fig. S1A in Text S1). However, we recovered viable transgenic parasites tagged in the PfE3w_Ap_ locus. Targeted integration of the cassette and transcription of PfE3w_Ap_-GFP was confirmed by PCR and RT-PCR (Fig. S1B–C in Text S1). Immunofluorescence assays showed PfE3w_Ap_-GFP to localize to the apicoplast (Fig. 1D). Finally, using an episomal expression vector, we found that the first 167 amino acids of PfE3c_Ap_ target a GFP reporter to the apicoplast (Fig. 1E).

Apicoplast proteins are often processed at the N-terminus removing a leader peptide [4]. We analyzed processing for TgE1_Ap_, TgE2_Ap_ and PfE2_Ap_ for which suitable reagents were available. TgE1_Ap_ produces the pattern typical for apicoplast proteins, two major bands likely corresponding to the precursor (heavier band) and mature protein (lighter band) Fig. 1F. Interestingly both TgE2_Ap_ and PfE2_Ap_ blots showed additional bands potentially arising from further post-translational modification (Fig. 1 G, H).

**Figure 2:**
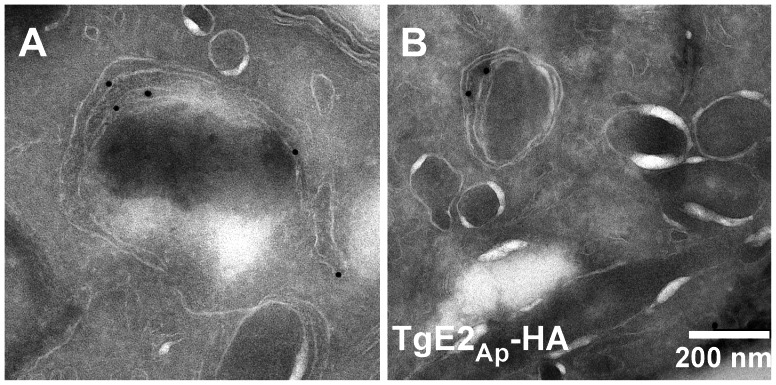
TgE2_Ap_ is localized to the periphery of the apicoplast. (**A, B**) Transmission electron micrograph of cryo-sections prepared from the TgE2_Ap_ -HA parasite line and labeled with anti-HA antibody and antiglobulin conjugated to colloidal gold. Gold beads are observed at the periphery of an organelle that is surrounded by four membranes.

While the immunofluorescence assays indicate apicoplast localization of the ubiquitylation enzymes, overlap with luminal markers is only partial (see enlarged insert in Fig. 1A). We fixed and processed TgE2_AP_-HA parasites for electron microscopy and incubated cryo-sections with an anti-HA antibody. Note that gold particles are found in the membranous periphery of the apicoplast (Fig. 2 and Fig. S4 in Text S1). This labeling is indistinguishable from that previously observed for the apicoplast ERAD-like proteins Der1_Ap_ and Cdc48_Ap_ [15] and the periplastid protein PPP1 [23]. We conclude that the apicoplast has a full complement of E1, E2 and E3 ubiquitylation enzymes localized to the periphery of the organelle, most likely the periplastid compartment as observed for the ERAD-like system in the diatom *Phaeodactylum* tricornitum [14], [18], [19].

### *In vitro* reconstitution of ubiquitylation using recombinant and native *Plasmodium* apicoplast enzymes

**Figure 3:**
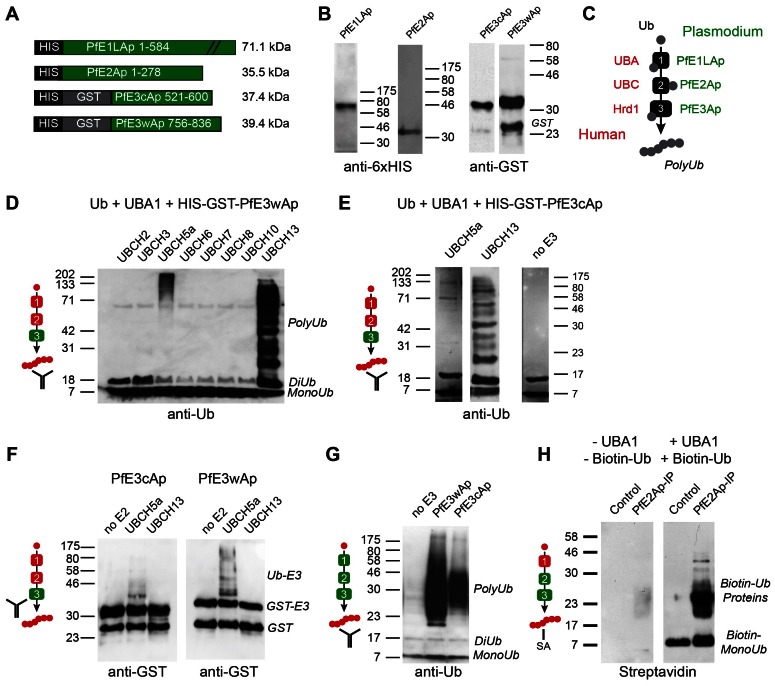
Recombinant *Plasmodium falciparum* apicoplast proteins show ubiquitin activation, conjugation and ligation activity. (**A**) Schematic maps of recombinant proteins indicating protein domains and purification tags used and the predicted molecular weight of the fusion protein (not to scale). (**B**) Western blot of recombinant proteins detected with antibodies to the indicated tag. (**C**) Schematic outline of the biochemical assay, ubiquitin is incubated with recombinant E1, E2 and E3 enzymes in the presence of ATP, human enzymes are shown in red, parasite enzymes in green (this color scheme is used as reference in panels **D**–**H**). Ubiquitylation is measured by Western blot detected polyubiquitin chains (PolyUb) either free, or linked to the E3 RING domain. The antibody symbol indicates the specific protein detected in each panel. PfE3w_Ap_ (**D**) or PfE3c_Ap_ (**E**) were incubated with human UBA1 and different UBCs as indicated. Note ubiquitylation using UBC5a (E3 associated higher molecular weight) and UBC13 (free Ub chains, only selected panels are shown in **E**). (**F**) Only the use of UBC5a results in ubiquitylation attached to PFE3_Ap_ visible as higher molecular weight species recognized by the antibody to the GST tag of E3. (**G**) Reconstitution of ubiquitylation using recombinant *Plasmodium* E1_Ap_ and E2_Ap_ and PfE3w_Ap_ or PfE3c_Ap_ respectively. (**H**) The presumptive E2/E3 complex immunoprecipitated from *P. falciparum* parasites using an antibody to PfE2_Ap_ shows ubiquitylation activity when incubated with biotinylated human ubiquitin and UBA1 (detected with Streptavidin, SA).

We next sought to establish whether the candidate apicoplast ubiquitylation system is capable of activating and ligating ubiquitin. We amplified or synthesized sequences encoding full length PfE1L_Ap_ and PfE2_Ap_, or the RING domains of PfE3w_Ap_ and PfE3c_Ap_ respectively, and engineered them to be expressed as recombinant fusion proteins carrying an N-terminal glutathione S-transferase (GST) and/or six-histidine (HIS) affinity tag. Proteins of the expected size could be purified for all four constructs (Fig. 3A, B). We established biochemical ubiquitylation assays using combinations of parasite enzymes and commercially available heterologous components (Fig. 3C, recombinant human factors are shown in red, *Plasmodium* enzymes in green). Enzymes were incubated with recombinant human ubiquitin in a buffer containing an ATP regenerating system. When analyzed by Western blot, ubiquitin chains can be detected as ladders of high molecular weight bands [24]. Among the numerous human ubiquitin-activating enzymes tested, UBCH5a and UBCH13 were found to be suitable partners for PfE3c_Ap_ and PfE3w_Ap_ leading to robust ubiquitylation. Note that this activity is strictly dependent on the recombinant parasite E3 and absent in controls (Fig. 3D, E). The pattern obtained differed between the two E2 enzymes and suggested ubiquitylation of the RING domain in the context of only UBCH5a, while interaction with UBCH13 appeared to produce free poly-ubiquitin. Variation of ubiquitylation pattern depending on the E2 partnered with the ligase is frequently observed [25]. To test this independently we probed the *in vitro* reaction with anti-GST antibody to visualize the E3 and its higher molecular weight ubiquitin adducts. Consistently, this revealed shifts in molecular weight of PfE3c_Ap_ and PfE3w_Ap_ only when incubated with UBCH5a (Fig. 3F) as free polyubiquitin is not detected in this assay format.

Next we tested whether ubiquitylation activity can be reconstituted entirely with parasite enzymes. When recombinant PfE1L_Ap_ and PfE2_Ap_ were incubated with ubiquitin alone (Fig. 3G, left lane), no ubiquitylation was detected. However, upon addition of recombinant E3 ligase PfE3w_Ap_ or PfE3c_Ap_, ubiquitylation was readily observed. Lastly we wished to evaluate the activity for native parasite enzymes. Among the reagents generated and tested in this study a custom-made antibody to PfE2_Ap_ was found suitable for immunoprecipitation under native conditions. Often the conjugating and ligating enzymes form a complex and can be co-precipitated and detected by their combined activity [26], [27]. We incubated pull down fractions from parasite lysates with recombinant human UBA1, and biotinylated-ubiquitin (using tagged ubiquitin enhances sensitivity and focuses the assay on only newly ubiquitylated proteins). We observed significant ubiquitylation that was dependent on the immunoprecipitate and UBA1 (Fig. 3H). Taken together our observations provide biochemical support for the notion that the apicoplast ERAD-like system is capable of mediating ubiquitylation.

### E2_Ap_ is required for parasite growth and protein import into the apicoplast

The apicoplast ERAD system has a critical role in protein import into the organelle [5], [18]. We tested whether ubiquitylation is a mechanistic requirement of this process by genetic ablation of the apicoplast ERAD-like ubiquitylation enzymes. We attempted disruption of the loci of PfE3c_Ap_, PfE3w_Ap_, and PfsUBA1. We isolated strains bearing drug marker insertions in the PfE3w_Ap_ gene and documented loss of associated transcription (Fig. S2B, S3C in Text S1). However, we also noted multiple genomic duplications in these strains complicating interpretation (Fig. S3D in Text S1). We did not obtain viable parasites with disrupted PfE3cAP or PfsUBA1 loci. This is consistent with a potentially essential role for these proteins, and we therefore turned to *T. gondii* where the construction of conditional mutants is feasible.

**Figure 4:**
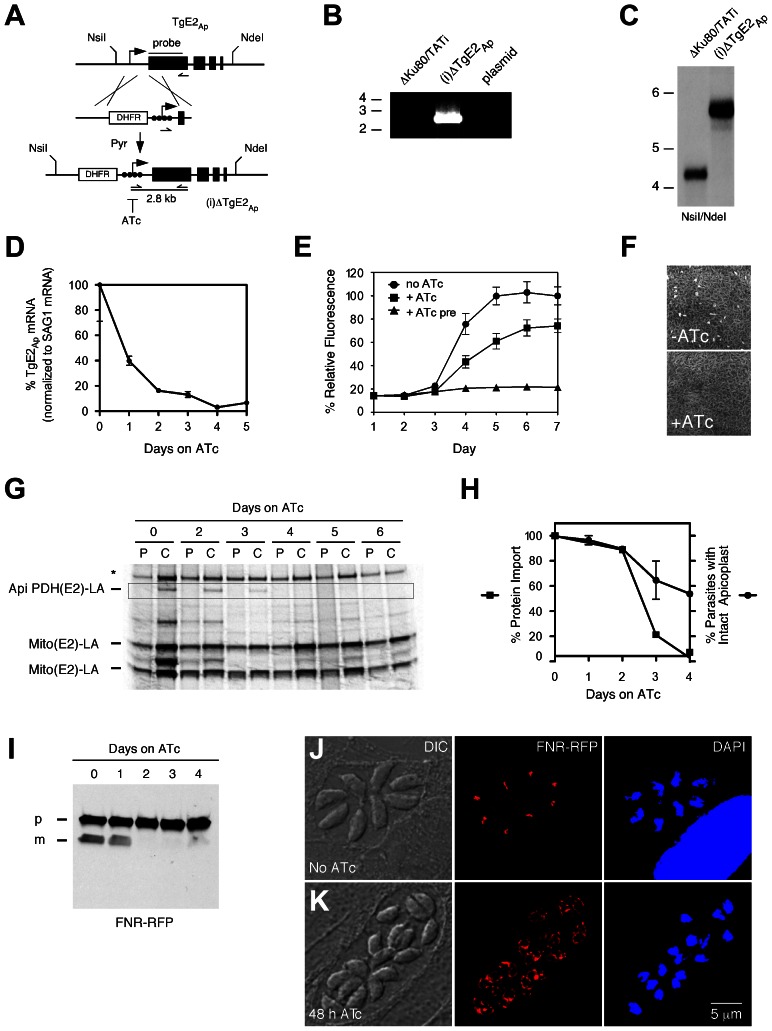
Genetic ablation of E2_Ap_ in *T. gondii* results in a block of apicoplast protein import and parasite growth. (**A**) The TgE2_Ap_ locus was modified homologous recombination and insertion of a DHFR selectable marker and a regulatable promoter (arrow, black circles indicate tet operator elements). Positions of diagnostic primers (half arrows) and probe (black line) are indicated. (**B**) PCR test for insertion, note that only the recombined locus is suitable to template the 2.8 kbp product. (**C**) Southern analysis hybridizing genomic DNA form parental (ΔKu80/TATI) or mutant ((i)ΔTgE2_Ap_) parasites with a probe derived from the first exon of TgE2_Ap_. (**D**) Real time PCR analysis of TgE2_Ap_ expression in (i)ΔTgE2_Ap_ parasites upon ATc treatment (normalized to the mRNA of the major surface protein SAG1, untreated control set to 100%, error bars show SD, n = 3) detects the expected DNA fragments. PCR analysis of clones shows integration of the promoter. (**E**) Growth of (i)ΔTgE2_Ap_ was measured by fluorescence (circles no drug, squares ATc, triangles 3 days of pretreatment with ATc prior to plate inoculation) and (**F**) plaque assay. (**G**) Apicoplast protein import in (i)ΔTgE2_Ap_ was measured by following PDH(E2) lipoylation ( [6] highlighted by box). Note loss of band upon ATc treatment and persistence of mitochondrial lipoylation (Mito (E2)-LA); Please note that there are two lipoylated proteins in the *T. gondii* mitochondrion [42], [43], * human PDH-E2. (**H**) Quantification of protein import (as measured in **G**, squares, data shown is representative of four experiments) and number of apicoplasts per parasite (circles, n = 3, SD shown) in (i)ΔTgE2_Ap_ over the course of ATc treatment. (**I**) Western blots probing FNR-RFP in (i)ΔTgE2Ap upon ATc treatment (p, precursor; m, mature protein). Note reduction in mature band upon treatment. Immunofluorescence assays showing (i)ΔTgE2Ap in the absence (**J**) or presence (**K**) of ATc. 38% of parasite vacuoles showed labeling outside the apicoplast, likely due to back up of cargo into the ER at 48 h of treatment (<3% in untreated parasites).

We engineered a parasite strain where the endogenous promoter of the TgE2_Ap_ gene was replaced by a regulatable promoter in the following referred to as (i)ΔTgE2_Ap_ (Fig. 4A, [23]). This was accomplished by double cross over in the *T. gondii* TATiΔKu80 background, a parasite line that favors homologous recombination and expresses a transactivator that can be modulated using anhydrotetracycline (ATc). Drug resistant parasite clones were tested by PCR and integration of the promoter was confirmed by Southern blot. We monitored the level of TgE2_Ap_ mRNA in response to ATc by quantitative PCR. Fig. 4D shows down-regulation of the transcript below 10% of its normal level at day four of ATc treatment. We asked whether loss of TgE2_Ap_ affects parasite growth and performed plaque and real-time fluorescence assays. Parasites grow normally in the absence of ATc indicated by formation of plaques, however in the presence of ATc, plaque formation is severely attenuated (Fig. 4F). Similarly, (i)ΔTgE2_Ap_ parasites show significant growth reduction in the fluorescence assay in the presence of ATc (Fig. 4E), preincubation of parasites in ATc abolished growth entirely. We conclude, that TgE2_Ap_ is critical for parasite growth.

We next tested the ability of (i)ΔTgE2_Ap_ parasites to import apicoplast proteins in the absence or presence of ATc and measured the import-dependent lipoylation of the apicoplast pyruvate dehydrogenase E2 subunit [6]. (i)ΔTgE2_Ap_ parasites were treated with ATc for different periods and pulse-labeled for one hour with [^35^S] methionine/cysteine. For the chase samples the radioactive isotope was removed, and cells were incubated for two additional hours in normal media. The samples were then used for immunoprecipitation with an anti-lipoic acid antibody followed by separation on SDS-PAGE. Treatment of cells with ATc for 2 days resulted in attenuation of import, leading to complete loss after 4 days (Fig. 4G, H). Lipoylation of two mitochondrial enzymes remained unaffected. We also monitored apicoplast loss, a frequent consequence of interference with apicoplast protein import [6], [15]. We observed a drop over time, but note that loss of import significantly precedes plastid loss. Loss of apicoplast protein import has also been shown to result in loss of leader peptide removal and backing up of precursor protein into the ER and other elements of the secretory pathway [6], [15], [28] We therefore measured the levels of precursor and processed mature form of the apicoplast reporter protein FNR-RFP [29].. We grew parasites for 0 to 4 days on ATc and performed Western blots using parasite protein extracts from each day. Probing these blots with an antibody against RFP revealed that precursor levels of FNR-RFP remained unchanged throughout the 4 days, while the mature protein was no longer detected after 2 days on ATc further supporting a strong import defect (Fig. 4I). We also monitored the localization of FNR-RFP in treated and untreated (i)ΔTgE2_Ap_ parasites by immunofluorescence assay. In untreated parasites FRN-RFP is restricted to the apicoplast (Fig. 4K). After 48 hours of ATc treatment 38% of parasite vacuoles also show significant labeling outside of the apicoplast surrounding the nucleus likely representing the ER (Fig. 4J, untreated TgE2_Ap_ or ATc treated wild type parasites showed such labeling in <3% of counted four cell vacuoles, n = 200). We conclude that apicoplast protein import is impaired in the absence of TgE2_Ap_.

### A conserved cysteine residue in the active site of TgE2_Ap_ is required for its function

**Figure 5:**
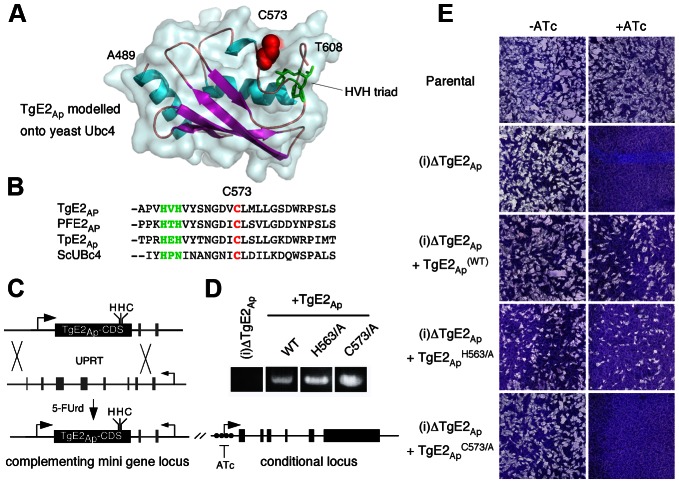
Active site residues are required for functional complementation of the TgE2_Ap_ null mutant. (**A**) MacPyMOL model of the secondary structure of TgE2_Ap_ (alignable residues A489-T608) using *S. cerevesiae* Ubc-4 enzyme as template and. Note three conserved α-helices (blue) and four β-strands (magenta). The active site cysteine residue is shown in red the HVH triad in green. (**B**) Multiple sequence alignment of the active site (cysteine, red: triad, green) from *P. falciparum*, *T. gondii*, the diatom *Thalassiosira pseudonana* and yeast (complete alignment shown in Fig. S3 in Text S1). (C) Wild type and point mutants of the TgE2_Ap_ coding sequence were introduced into the UPRT locus of TgE2_Ap_ (**C**) under 5FUrd selection, correct insertion was established by PCR (**D**) and phenotypic complementation was assessed by plaque assay in the presence or absence of ATc (**E**).

Apicoplast ubiquitylation enzymes are capable of synthesizing ubiquitin chains in vitro, but is this activity required *in vivo*? To test this we established a complementation assay. The coding sequence of the TgE2_Ap_ gene driven by a constitutive promoter was introduced into the uracil-phosphoribosyltransferase (UPRT) locus of the (i)ΔTgE2_Ap_ mutant (Fig. 5C). Parasites were selected for the loss of UPRT activity using 5-fluorodeoxyuridine [30] and a clonal cell line that now constitutively expressed a second copy of TgE2_Ap_ in the conditional knock down background was isolated. We confirmed correct integration by PCR (Fig. 5D). We tested the ability of this strain to form plaques when expression from the native locus is ablated by ATc treatment, and found that genetic complementation fully rescued growth (Fig. 5E).

Multiple sequence alignment of TgE2_Ap_ and E2 enzymes from a wide range of eukaryotes showed that TgE2_Ap_ shares conserved features, reported earlier to be critical for this class of enzymes. We therefore modelled the C-terminal domain of TgE2_Ap_ onto the structure of UBC4, a well characterize yeast ubiquitin conjugating enzyme [31]. Multiple sequence alignment and homology modelling identified C573 as the presumptive active site cysteine (Fig. 5A, B, see Fig. S3 in Text S1). Most E2 enzymes possess a signature HPN triad proximal to the active site cysteine [32]. The histidine has been previously suggested to be dispensable for E2-catalyzed ubiquitylation, but is important for the folding of the active site in other systems [33]. The asparagine residue on the other hand was consistently found to be important for RING-E3/E2-dependent ubiquitin conjugation [34]. A conserved HXH triad is found at this position in apicomplexans (Fig. 5B). We engineered a series of point mutants in TgE2_Ap_ replacing C573, H563, and H565 with alanine respectively. These genes were then introduced into the (i)ΔTgE2_Ap_ mutant as described above and tested for their ability to complement loss of TgE2_Ap_ upon ATc treatment using plaque assay. Expression of the H563A point mutant fully complemented loss of native TgE2_Ap_ (Fig. 5E) and parasites now grow even in the presence of ATc. In contrast, despite numerous attempts we were unable to establish a stable parasite line expressing H565A, which may suggest dominant effects of this mutation. We were able to isolate mutants expressing C573A, however these strains show no complementation, and are still fully susceptible to ATc treatment (Fig. 5E). We conclude that enzymatic activity is a requirement for TgE2_Ap_ function *in vivo* and that C573 and H565 residues are critical for the function of the enzyme while H563 is likely dispensable.

## Discussion

Endosymbiosis is a key evolutionary mechanism underlying the emergence and diversification of eukaryotes – in particular for photosynthetic eukaryotes. The acquisition of a eukaryotic red algal symbiont led to the chromalveolates, a large super-phylum of tremendous ecological diversity that includes apicomplexan parasites. The descendent of the algal symbiont, the apicoplast, maintains a highly compartmentalized organization, and nuclear encoded proteins have to overcome four membranes on their journey to the stroma. An apicoplast-localized ERAD-like system appears to play an important role in apicoplast protein import. Recent reports have identified and characterized components of this ERAD–like system in different algal and parasite species [7], [10], [12], [13], [14], [15]. In this study we provide significant biochemical and genetic evidence for the hypothesis that an apicoplast localized ubiquitylation cascade is an essential element of this protein import system. We identify apicoplast ubiquitin activating, conjugating and ligating enzymes in two important apicomplexan parasites, *P. falciparum* and *T. gondii*. We show *in vitro* and *in vivo* that these proteins have conserved biochemical activities and are capable of ubiquitin transfer. Finally, in genetic studies, we show that TgE2_Ap_, for which we were able to isolate a conditional mutant, is essential for apicoplast protein import, organellar maintenance and parasite growth. Overall these observations support a direct mechanistic role of ubiquitylation in protein translocation independent of ubiquitin's function in proteasomal degradation [11]. The classical ERAD system is believed to recognize and respond to the folding state of secretory proteins. Interestingly, recent studies show that the transit peptide of apicoplast proteins is primarily unstructured and that this conformation may be critical for proper transport to the organelle [35]. This model would need a distinguishing element to avoid elimination of apicoplast proteins by the classical ERAD. Specific chaperone sets could potentially provide such specificity, but remain to be discovered. A recent study in *Arabidopsis* has identified a role for ubiquitylation also in primary plastids, however this role appears to be distinct from secondary plastids. In this case ubiquitylation results in degradation of the components of the TOC complex and is thought to more globally regulate chloroplast biogenesis during plant development [36].

The identity of the apicoplast ubiquitin or ubiquitin-like modifier remains a significant unresolved question. Our results demonstrate that apicoplast enzymes are capable of acting on archetypical ubiquitin (recombinant human protein), studies in *P. tricornitum* show similar activity for a E3 ligase found in the diatom secondary chloroplast [18]. However whether the apicoplast system actually utilizes ubiquitin *in vivo* remains to be established. As shown in Fig. 1G and H Western blots for TgE2_Ap_ and PfE2_Ap_ show additional bands. It is conceivable that these bands represent ubiquitin or a ubiquitin-like protein covalently bound to the active site of the apicoplast localized E2. However we note that, for TgE2_A_, none of the bands was affected by reduction of the protein or point mutation of the active site cysteine (data not shown). Alternatively this may indicate an ubiquitin-like protein bound to a residue different from the active site of the enzyme or multiple processing steps as have been observed for some apicoplast membrane proteins [37]. Our efforts to demonstrate ubiquitin bound to apicoplast ubiquitylation enzymes purified from *P. falciparum* or *T. gondii* so far did not result in robust detection (using either antibodies or mass spectrometry, data not shown). Furthermore ubiquitylation of plastid-bound cargo proteins is not readily observed in apicomplexans or diatoms. A reasonable candidate for which apicoplast localization has been suggested [12] is an atypical, large ubiquitin-like protein (PF08_0067). Curiously, this protein lacks the di-glycine motif typically required for the formation of the isopeptide bond and a homolog has yet to be identified in the *Toxoplasma* genome. Similarly, plastid ubiquitin candidates from algae show lack of sequence motifs typically required for polyubiquitylation [19]. It is conceivable that this ubiquitin-like protein could be processed and/or ligated in a novel fashion that does not depend on a di-glycine sequence. Alternatively, its function may resemble that of the HERP protein in the classical ERAD pathway. Like PF08_0067, HERP has an ubiquitin-like domain at the N-terminus followed by transmembrane domains at the C-terminus [38]. HERP is believed to interact with HRD1 and to regulate the ubiquitylation activity of the ERAD translocon in response to folding stress [39]. In that case PF08_0067 is likely not the main substrate for the apicoplast ubiquitylation system and the modifier is yet to be discovered.

Studying the apicoplast ubiquitin faces technical obstacles that so far prevented direct tagging of the candidate ubiquitin and subsequent detection of modified cargo. There are several strong candidates for plastid-localized deubiquitylation enzyme in apicomplexans and diatoms (Table S1 in Text S1, [13], [18]). The activity of these enzymes may dramatically shorten the time ubiquitin remains on proteins and thus prevent the robust detection of ubiquitin adducts [22]. Isolation of mutants lacking apicoplast deubiquitylation might allow testing of this hypothesis and potentially lead to accumulation of modified cargo proteins. While a number of mechanistic details of the apicoplast ubiquitylation system remain to be elucidated, we demonstrate that the system is essential to the organelle and the parasite. Building on a longstanding effort to target ubiquitylation for the development of anti-cancer drugs [40] may potentially lead to new anti-parasitic compounds in the future.

## Materials and Methods

*P. falciparum* strains 3D7, D10_ACP-(leader)-GFP (MR4, MRA568) and derivatives were cultured in human O+ red blood cells [41]. *T. gondii* RH strain parasites and derivatives were propagated in human fibroblasts and genetically modified as described [6], [15].

For *in vitro* ubiquitylation assays recombinant *P. falciparum* enzymes were incubated with recombinant human or parasite factors. Typically 50–200 µM recombinant ubiquitin, 0.05–0.2 µM E1 enzyme, 1–5 µM E2 enzymes, and 1–12.5 µM of E3 ligases were incubated in 50 mM Tris-HCl, pH 7.4, 1 mM DTT in presence of a re-energizing system (BostonBiochem) containing the ATP and ATP regenerating enzymes to recycle hydrolyzed ATP needed for the assay, for 2 hours at 37°C followed by SDS-PAGE and immunoblotting.

*Ex vivo* ubiquitylation assays were performed by lysing 3D7 *P. falciparum* in 20 mM HEPES pH 7.9, 10 mM KCl, 1 mM EDTA, 1 mM EGTA, 1 mM DTT, 0.5 mM AEBSF (Fisher Scientific), 0.65% Igepal v/v, and protease inhibitor cocktail (Roche), or 20 mM HEPES pH 7.9, 0.1 M NaCl, 0.1 mM EDTA, 0.1 mM EGTA, 1.5 mM MgCl2, 1 mM DTT, 1 mM AEBSF and protease inhibitor cocktail (Roche). Supernatants were pooled and proteins were precipitated using the indicated antibodies and magnetic Protein A beads. Proteins bound to beads were mixed with re-energizing buffer, 0.5 µg/µl biotin-conjugated ubiquitin, 5 mM AEBSF and protease inhibitor cocktail. Reactions were incubated at 30°C with gentle agitation for two hours. Samples were eluted with 4× Laemmli buffer and analysed using biotin affinity blots. Human recombinant UBE1 and UBC enzymes, E3 ligases biotin conjugated ubiquitin and re-energizing buffer used in these assays were purchased from Boston Biochem.

*T. gondii* gene models were tested by 5′- and 3′-RACE. Note that additional exons were identified for TgE2_Ap_ (see genbank JX431938 for correct sequence). A conditional TgE2_Ap_ knock-out was generated by exchanging the native promoter for the tetracycline inducible t7s4 promoter in the TATiΔKu80 parasite background. The targeting construct used 1.2 kb up- and 1.5 kb downstream of the TgE2_Ap_ start codon introduced into vector pDT7S4. Linearized plasmid was transfected into the parental strain followed by pyrimethamine selection. To complement the knock-out, a TgE2_Ap_ minigene was inserted into the UPRT locus under the control of a constitutive sag1 promoter. Transgenics were isolated in 5 µM 5-FUDR and identified by PCR. Parasite growth was measured by fluorescence and plaque assay in the presence and absence of 0.5 µm anhydrotetracycline (ATc). Please refer to the supplement materials for a more detailed description of materials and methods used in this study (including a table of all primers).

## Supporting Information

Text S1 **Provides supplemental** **materials and methods** **used for the Bioinformatic analysis.** We also provides additional information for the *T. gondii* plasmids, cell line and cell culture used; the apicoplast protein import assay; the antibody based assay; the cloning and purification of recombinant *P. falciparum* proteins and finally the Phylogenetic analysis and homology modelling of *T. gondii* TgE2_Ap_. Figure S1 presents the 3×HA tagging strategy used for PfE3c_Ap_. Figure S2 shows the gene disruption strategies used for PfE1L_Ap_, PfE3c_Ap_ (PFC0740c) and PfE3w_Ap_. Figure S3 shows the multiple sequence alignment for E2 enzymes using MUSCLE. Figure S4 shows additional cyro-electonmicroscopic images of TgE2_Ap_. Table S1 presents the apicoplast candidates for E1, E2, E3 and Dub enzymes in both *P. falciparum* and *T. gondii* and lastly table S2 present a list of primers used for the cloning of recombinant proteins. **Figure S1**. (A) 3×HA tagging of PfE3c_Ap_ (PFC0740c) strategy is shown in the left panel. V-H indicates primer pairs that amplify only if there is targeted integration of the 3×HA plasmid to the PfE3c_Ap_ gene. After recovery, there was a diminishment of V-H PCR products over time (right panel), indicating that transfected strains exhibited a delayed-death effect of properly integrated vectors, leaving only recovered strains with non-integrated plasmids. The number of weeks (W1, W6, W12) starts from the time we observed recovered strains. (B) 3D7 parasite strains were transfected with plasmids that had a GFP fused to the c-terminus of the PfE3w_Ap_ (PFC0510w) for targeted integration by homologous recombination. Transfected strains (PFC0510w-GFP) were screened by PCR, where primers pairs (Ver) only amplified a product if proper integration had taken place. (C) RT-PCR reveals that GFP fused to the c-terminal of PFC0510w is being transcribed in the PFC0510w-GFP strains. **Figure S2**. (A) Gene disruption strategy of PfE1L_Ap_ (PF13_0182), PfE3c_Ap_ (PFC0740c) and PfE3w_Ap_ (PFC0510w). PCR of transfected strains with disruption vectors show that only PFC0510w was successfully disrupted. (B) RT-PCR of sub-cloned PFC0510w disrupted strains show no transcription of the RING domain of PFC0510w. (C) CGH microarray analysis show that the PFC0510w gene disruption vector was able to integrate into the genome of the transfected strain. **Figure S3**. Multiple sequence alignment for E2 enzymes using MUSCLE. **Figure S4**. Additional cyro-electonmicroscopic images of TgE2_Ap_. Sections were incubated with anti-HA antibody and gold conjugated anti-immunoglobulin as detailed in Figure 2. **Table S1**. Apicoplast candidates for E1, E2, E3 and Dub enzymes in both *P. falciparum* and *T. gondii*. **Table S2**. List of primers used for the cloning of all recombinant proteins.
(DOC)
