# Supplementary material for: An Apicoplast Localized Ubiquitylation System Is Required for the Import of Nuclear-encoded Plastid Proteins
Source: PLoS Pathog. 2013 Jun 13;9(6):e1003426. doi: 10.1371/journal.ppat.1003426 (PMC3681736; doi:10.1371/journal.ppat.1003426)
Supplement: Text S1 — Provides supplemental materials and methods used for the Bioinformatic analysis. We also provides additional information for the T. gondii plasmids, cell line and cell culture used; the apicoplast protein import assay; the antibody based assay; the cloning and purification of recombinant P. falciparum proteins and finally the Phylogenetic analysis and homology modelling of T. gondii TgE2Ap. Figure S1 presents the 3×HA tagging strategy used for PfE3cAp. Figure S2 shows the gene disruption strategies used for PfE1LAp, PfE3cAp (PFC0740c) and PfE3wAp. Figure S3 shows the multiple sequence alignment for E2 enzymes using MUSCLE. Figure S4 shows additional cyro-electonmicroscopic images of TgE2Ap. Table S1 presents the apicoplast candidates for E1, E2, E3 and Dub enzymes in both P. falciparum and T. gondii and lastly table S2 present a list of primers used for the cloning of recombinant proteins. Figure S1. (A) 3×HA tagging of PfE3cAp (PFC0740c) strategy is shown in the left panel. V-H indicates primer pairs that amplify only if there is targeted integration of the 3×HA plasmid to the PfE3cAp gene. After recovery, there was a diminishment of V-H PCR products over time (right panel), indicating that transfected strains exhibited a delayed-death effect of properly integrated vectors, leaving only recovered strains with non-integrated plasmids. The number of weeks (W1, W6, W12) starts from the time we observed recovered strains. (B) 3D7 parasite strains were transfected with plasmids that had a GFP fused to the c-terminus of the PfE3wAp (PFC0510w) for targeted integration by homologous recombination. Transfected strains (PFC0510w-GFP) were screened by PCR, where primers pairs (Ver) only amplified a product if proper integration had taken place. (C) RT-PCR reveals that GFP fused to the c-terminal of PFC0510w is being transcribed in the PFC0510w-GFP strains. Figure S2. (A) Gene disruption strategy of PfE1LAp (PF13_0182), PfE3cAp (PFC0740c) and PfE3wAp (PFC0510w). PCR of transfe [file ppat.1003426.s001.doc]

**Supplemental Information**

**Supplemental Materials and Methods.**

***Bioinformatic analysis***

***Proteins and domain motifs datasets***

The translated genome of *P. falciparum* v8.0 was obtained from PlasmoDB ([www.plasmodb.org](http://www.plasmodb.org/)). A second *P. falciparum* proteins dataset was built and consists of 473 genes which products are predicted to be apicoplast-targeted by PlasmoAP (available on PlasmoDB). Twenty-seven hidden Markov models (HMM) of domain motifs commonly found within ubiquitylating proteins were obtained from the PFAM database [1] (Pfam accession numbers PF04110, PF00888, PF05903, PF00646, PF00632, PF02099, PF02991. PF01398, PF02338, PF01088, PF02902, PF03416, PF00519, PF07525, PF00899, PF04564, PF09358, PF02134, PF00240, PF00443, PF03671, PF00179, PF09138, PF08325, PF00097, PF08746, PF02891).

***Pfam domain search using probabilistic pattern recognition***

The HMM search component of the HMMER 3.0 package was used to identify proteins that carry ubiquitylation-related domain motifs by iterative probabilistic pattern recognition [2,3]. Results were manually curated by visual inspection of protein sequence alignments using hmmalign and mview [4] performed on the web bioinformatics portal Mobyle [5]. Analyses were performed on both the *P. falciparum* translated genome and the 473 candidate apicoplast-targeted proteins to identify ubiquitylating proteins.

***Identification of apicoplast-targeted ubiquitylating candidate proteins***

The N-terminal portion of the ubiquitylating proteins identified by HMM search was further analyzed for the presence of targeting motifs using PATS v1.2.1N [6], PlasmoAP [7], TargetP v1.1 [8] (with the prediction of the signal peptide cleavage site turned on [9], ChloroP v1.1 [10], and SignalP3.0 [11]. Homologues were identified in the NCBI non-redundant protein database using BLASTP v2.2.24+ [12] and aligned using MUSCLE [13] and T-COFFEE [14].

***Protein signature domain analysis and domain architecture***

The domain architecture of the selected candidates was analyzed using the InterProScan Sequence Search from the InterPro database [15]. In addition, transmembrane domains were predicted by TMHMM v2.0c. Scenarios for spatial organization of the transmembrane domains were built using TMHMMfix [16].

***T. gondii plasmids, cell line and cell culture***

The TgE2_Ap_-HA expression vector was constructed by cloning the full-length cDNA of TgE2_Ap_ into BglII/ AvrII sites of the pDt7s4H vector. The resulting plasmid was transfected into *T. gondii* TATi strain as previously described [17]. TgE2_Ap_-HA expressing parasites were selected by propagation under drug pressure with 1μM pyrimethamine. To engineer an endogenously tagged TgE1_Ap_ strain, 1.5kb of TgE1_Ap_ genomic DNA upstream of the stop codon was cloned into the pLIC-HA-CAT vector by ligation independent cloning as described by Huynh and colleagues [18]. The final construct was linearized and transfected in ΔKu80 parasites [18]. TgE1_Ap_-HA expressing parasites clones were obtained after chloramphenicol drug selection. Integration of the tag and expression of the chimeric protein was confirmed by PCR analysis (not shown here) and Immunofluorescence assay with anti-HA antibody.

For targeted insertion of an inducible promoter at the endogenous locus of TgE2_Ap_, 1.5Kb of genomic sequence from the start of the gene was PCR amplified. This fragment was cloned into BglII/ AvrII sites of the pDt7s4H vector. Next a 1kb flank immediately upstream of the gene (putative promoter region) was PCR amplified with synthetic NdeI restriction sites and cloned into equivalent sites of the above vector. The resulting construct was linearized by digestion with AvrII and transfected into pyrimethamine-sensitive tetracycline transactivator expressing ΔKu80 parasites (ΔKu80-TATi) [19]. The pyrimethamine-resistant parasites were cloned parasites by limiting dilution and successful modification of the genetic locus was confirmed in the clones by PCR analysis. Single site integration into genomic DNA was confirmed by Southern blot hybridization as described in [20]. Briefly, gDNA was extracted using the Qiagen DNA-easy kit, from ΔKu80-TATi and (i)ΔTgE2_Ap_ cell lines. The DNA was digested overnight with NsiI/NdeI restriction enzyme. The digested DNA was separated on 1% agarose gel, blotted on nitrocellulose membrane and probed with radiolabeled TgE2_Ap_ cDNA amplified by PCR as the probe.

For complementation of the TgE2_Ap_, TgE2_Ap_ minigene versions (wild type and point mutant) were expressed in the (i)ΔTgE2_Ap_ cell line under the control of the constitutive sag1 promoter. The cDNA was cloned into NsiI/NheI restriction sites downstream of the sag1 promoter in a plasmid containing 2 kb flanking regions upstream and downstream of the uracil phosphoribosyl transferase (UPRT) gene. These flanks were used to insert the transgene into the endogenous UPRT locus by double homologous recombination. Point mutants were generated with the Quik-Change II XL Site-Directed Mutagenesis Kit (Stratagene) using the plasmid described above as template [21]. Different variants of this construct were then transfected into the inducible (i)ΔTgE2_Ap_ cell line and selected with 5μM 5-FUDR after 48 hrs of transfection.

***Apicoplast protein import assay***

Apicoplast import assays were performed as previously described [20,22]. Briefly, infected host cells were starved for one hour in cysteine and methionine-free Dulbecco’s Modified Eagles medium supplemented with 1% fetal bovine serum and antibiotics. Infected host cells were then radiolabelled with 100 mCi/mL of ^35^S methionine and cysteine (MP biomedicals) for one hour. Cells were either harvested (pulse) or washed twice with 10 ml of parasite growth medium, and incubated in 10 ml parasite growth medium for 2 to 8 hours before harvesting. Proteins of interest were purified by affinity purification and separated by SDS-PAGE as described above. Gels were dried and bands were visualised by autoradiography or using a Storm 860 PhosphorImager (GE Healthcare). Band intensities were quantified using Image Quant TL software (GE Healthcare).

For immunoprecipitations, parasites were lysed from host cells by passage through a 26 gauge needle, and pelleted by centrifugation at 1500g for 10 minutes. Pellets were washed in PBS then lysed for 30 minutes on ice in immunoprecipitation lysis buffer (50 mM Tris-HCl, pH 8.0, 150 mM NaCl, 1 % (v/v) noniodet P-40 substitute (Fluka), 0.5 % (w/v) sodium deoxycholate, 0.1 % (w/v) sodium dodecyl sulphate, 2 mM EDTA) supplemented with protease inhibitors (Complete protease inhibitor cocktail, Roche Applied Science). Samples were centrifuged to remove insoluble material. Proteins-of-interest were purified by immunoprecipitation using antibodies bound specifically to protein A-sepharose CL-4B beads (GE Healthcare). Samples were pre-cleared by incubation in 30 to 40 mls of a 50 % slurry of Protein A-Sepharose CL-4B beads. Anti-lipoic acid (Calbiochem) was bound to protein A-sepharose CL-4B beads for one hour at 4^o^C before addition to pre-cleared lysates. All samples were incubated overnight at 4^o^C then washed 4 times in immunoprecipitation wash buffer (50 mM Tris-HCl, pH 8.0, 150 mM NaCl, 1 % (v/v) noniodet P-40 substitute, 0.5 % (w/v) sodium deoxycholate, 0.25 % w/v bovine serum albumin, 2 mM EDTA) and twice in PBS. Samples were eluted by boiling in non-reducing sample buffer prior to separation by SDS-PAGE as described above.

***Antibody based assays***

Western blotting, pulse-chase analyses, immunoprecipitations, and immunofluorescence assays, using *T. gondii* were performed as described [20,22] using the following antibodies: rat anti-HA (Roche), rabbit anti-*Tg*Cpn60 [20], rabbit anti-lipoic acid (Calbiochem), anti-Mal13P1.227 (1:100), goat anti-GST antibodies (1:5000; GE Healthcare), mouse anti-HIS antibodies (1:2500; Millipore), anti-ubiquitin antibodies (1:2500; Upstate), streptavidin conjugated with peroxidase (1:10,000; Jackson Immunoresearch). Secondary antibodies used for immunofluorescence assays were goat anti-rabbit Alexa Fluor 546 (1:500), goat anti-rat Alexa Fluor 488, goat anti-rabbit IgG Alexa Fluor® 488 (1:100), donkey anti-rabbit IgG Alexa Fluor® 568 (1:100). For western blotting the following horseradish peroxidase (HRP) conjugated secondary antibodies were used: donkey anti-goat antibodies (1:20,000; Jackson Immuno research), goat anti-mouse antibodies (1:10,000; BioRad), goat anti-rabbit antibodies (1:5000; Pierce) and goat anti-rat antibodies (1:5000; Pierce). For quantitative analysis of apicoplast biogenesis defects (i)ΔTgE2_Ap_ parasites were grown for 0-4 days on ATc and stained with Cpn60 antibody. One hundred four-cell vacuoles were imaged at each time point. We graphed the percentage of vacuoles at each time point where every parasite in that vacuole contained an apicoplast. For analysis of cargo retention en-route to the apicoplast, we treated FNR-RFP expressing (i)ΔTgE2_Ap_ parasites with/ without ATc for 48 hours. Samples were scored for ER retention using the following criteria. Signal outside the apicoplast had to be obviously apparent by signal throughout the ER or punctate appearance with the beginning of diffusing signal within the ER. For each treatment, 200 parasite vacuoles were scored and counted.

*P. falciparum* immunofluorescence images were prepared as described previously [23], observed with the Olympus BX40 microscope using a 100x objective lens (UPlanFI) and captured by the CoolSNAP cf (Photometrics) camera using Metavue. Images were merged and adjusted using ImageJ software. *T. gondii* immunofluorescence analysis were carried out as described in [20]. Images were collected on an Applied Precision Delta Vision or a Leica DIRBE microscope and images were deconvolved and adjusted for contrast using Softworx and Openlab software.

For TgE2_Ap_ cryo-electron microscopy, infected cells were fixed and processed in 4% paraformaldehyde/0.05% glutaraldehyde (Polysciences Inc.) in 100mM PIPES buffer. Samples were then embedded in 10% gelatin and infiltrated overnight with 2.3M sucrose/20% polyvinyl pyrrolidone in PIPES at 4°C. Samples were frozen in liquid nitrogen and sectioned with a cryo-ultramicrotome. Sections were probed with the indicated primary antibodies followed by the appropriate secondary antibody conjugated to 12 or 18 nm colloidal gold, stained with uranyl acetate/methylcellulose, and analyzed by transmission EM as described previously in [24].

A vector containing the active domain of the PfE2_Ap_ was kindly gifted by Dr. R. Hui [25] and was used to generate a PfE2_Ap_ (Mal13P1.227) antibody produced in rabbit (Genscript).

***Cloning and purification of recombinant P. falciparum proteins***

To express the RING domains of PfE3c_Ap_ (PFC0740c) and PfE3w_Ap_ (PFC0510w), codon optimized (Genscript) fragments were cloned into pGS-21a (Genscript), which contains both GST and 6xHIS tags. GST purification was performed with glutathione agarose (Sigma). Bound proteins were washed three times with GST wash buffer (25mM Tris-HCl, pH 7.5, 300mM NaCl and 1% Triton X-100). GST-tagged proteins were then eluted with GST elution buffer (25mM Tris-HCl, pH 7.5, 150mM NaCl, 15mM reduced glutathione and 0.01% Triton X-100 and 40% (v/v) glycerol). Anti-GST immunoblots were probed with goat anti-GST antibodies (1:5000; GE Healthcare) and donkey anti-goat antibodies conjugated to horseradish peroxidase (HRP) (1:20,000; Jackson Immuno research).

6xHIS tagged PfE2_Ap_ (Mal13P1.227) and PfE1L_Ap_ (PF13_0334), recombinant proteins were cloned into a modified version of PGS21a (PGS-21aHIS). HIS-tagged proteins were absorbed to Ni-NTA beads (Qiagen) and subsequently washed several times with a solution containing 25mM Tris-HCL pH 7.5, 500mM NaCl, 30mM imidazole and 5% glycerol. Purified proteins were eluted with 25mM Tris-HCl pH 7.5, 500mM NaCl, 250mM imidazole and 5% glycerol. Anti-HIS immunoblots were probed with mouse anti-HIS antibodies (1:2500; Millipore) and goat anti-mouse antibodies conjugated to horseradish peroxidase (HRP) (1:10,000; BioRad). Please refer to Supplemental Table 2 for a list of primers used for the cloning of recombinant proteins.

**Phylogenetic analysis and homology modelling of *T. gondii* TgE2_Ap_**

E2 homologues included in phylogenetic anyalsis were ([genbank accession number], [*joint genome institute accession numbers]) from *Cyanidioschyzon**merolae* [CMQ038], *Emiliania huxeyli* [104731*]], *Emiliania huxyeli* [444197 *], *Plasmodium falciparum* [XP_001351133], *Plasmodium falciparum* apicoplast E2 [[XP_001350169.1](http://www.ncbi.nlm.nih.gov/protein/124513626?report=genbank&log$=prottop&blast_rank=1&RID=ZEAGMPH0011)], *Babesia bovis* [XP_001609155.1], *Babesia bovis* apicoplast E2 [XP_001610192],*Theileria parva* [XP_766253.1], *Theileria parva* apicoplast E2 [XP_765206.1], *Cryptosporidium parvum* [XP_001388391.1], *Toxoplasma* gondii [[XP_002371470.1](http://www.ncbi.nlm.nih.gov/protein/237844345?report=genbank&log$=prottop&blast_rank=1&RID=ZEBPPCC601S)], *Toxoplasma* gondii apicoplast E2 [TGME49_295990, genbank accession number JX431938], *Phaeodactylum tricornitum* [XP_002178369.1], *Thalassiosira pseudonana* [XP_002291866.1], *Arabidopsis thaliana* [NP_565110.1], *Homo sapiens* [AAC41750.1], *Chlamydomonas reinhardtii* [XP_001694965.1], *Paramecium tetraurelia* [XP_001430446.1], *Tetrahymena thermophila* [XP_001018543.1], *Pisum sativum* [AAA64427.1], *Drosophila melanogaster* [CAA72184.1], *Mus musculus* [AAB05772.1], *Oryza sativa* [AAB02168.1], Caenorhabditis *elegans* [AAB25489.2], *Schizosaccharomyces pombe* [NP_594929.1], *Saccharomyces cerevisiae* [CAA58975.1], *Leishmania major* [XP_001686040.1], *Entamoeba histolytica* [XP_001913876.1], *Guillardia theta* Nucleomorph [AAK39779.1]. E2 protein sequences from the above eukaryote taxa were used to generate a multi-sequence alignment using ClustalX version 2.0.

Full length verified amino acid sequence of TgE2_Ap_ was used to identify homologous sequences with gapped BLAST query 2.2.16 and Inter-ProScan provided by Swiss Expasy. The most homologous template, with greatest similarity sequence, was *Saccharomyces cerevisiae* UBC4 gene, with protein data bank code 1qcqA [26] (PDB) taken from Research Collaboratory for Structural Bioinformatics (RCSB) [www.pdb.org](http://www.pdb.org/) [27]. The sequence of TgE2_Ap_ protein was submitted to SWISS-Model for homology modeling in the automated mode. Modeling was performed using the coordinates deposited for the crystal structures of *Saccharomyces cerevisiae* UBC4 (1qcqA.pdb) as template. Protein structures were visualized with MacPyMOL (www.pymol.org). 119 residues Ala489-Thre608 were successfully modeled after yeast UBC-4 protein. The final structure was further checked by VERIFY-3D graph.

**Table S1.**

| **Putative Function** | | ***P. falciparum*** | | ***T. gondii*** | **p-value** |
| --- | --- | --- | --- | --- | --- |
| ubiquitin | | PF08_0067 | | TGME49_289750 | 0.97* |
| E1 ubiquitin-activating enzyme | | PF13_0182 | | TGME49_314890 | 1.00E-23 |
| E1 ubiquitin-activating enzyme | | PF13_0344 | | TGME49_212100 | 1.10E-27 |
| E2 ubiquitin-conjugating enzyme | | MAL13P1.227 | | TGME49_295990 | 0.084 |
| E3 ring zf-C3H4 ubiquitin ligase | | PFC0740c  (PfE3c_Ap_) | | TGME49_226740 | 1.20E-17 |
| E3 ring zf-C3H4 ubiquitin ligase | | PFC0510w  (PfE3w_Ap_) | | TGME49_304460 | 0.00019 |
| OTU deubiquitinase | | PF10_0308 | | TGME49_260510** |  |
| mov34 deubiquitinase | | PF10_0233 | | N/D |  |
| mov34 deubiquitinase | | MAL8P1.126 | | N/D |  |
|  | * Using only the ubiquitin-like domain of PF08_0067 | |  | | |
|  | **from Ponts et al 2008 | |  | | |

**Table S2.**

| **Cloning Purpose** | **Amplied product** | **Forward Primer** | **Reverse Primers** | **Enzymes used** |
| --- | --- | --- | --- | --- |
| HA tagging | TgE2_Ap_ (Full length cDNA) | GATCagatctAAAATGGGGCCACGGTGGGCGGTGAGGTG | GATCcctaggCACCTTGTCGTCGTGGTACAGGAACTG | BglII, AvrII |
| Gene disruption | TgE2_Ap_(5'flank) | GATCcatatgCAGACTTGTTCCCTCCGTTGCCTT | GATCcatatgTGTCTTCCTTGTTCGTTGCCATAGC | NdeI, NdeI |
| Gene disruption | TgE2_Ap_(3'flank) | GATCagatctAAAATGGGGCCACGGTGGGCGGTGAGGTG | GATCcctaggCCTTCTGAATGCGGTAATTCGCATG | BglII, AvrII |
| Complementation | TgE2_Ap_ (Full length cDNA) | GATCatgcatAAAATGGGGCCACGGTGGGCGGTGAGGTG | GATCgctagcCACCTTGTCGTCGTGGTACAGGAACTG | NsiI, NheI |
| TgE2ApC573A point mutant | sag1-TgE2_Ap_-UPRT plasmid | TCTAACGGAGATGTCGCGCTAAATCTTCTCGGC | GCCGAGAAGATTTAGCGCGACATCTCCGTTAGA |  |
| TgE2ApH563A point mutant | sag1-TgE2_Ap_-UPRT plasmid | CAACCGGCTCCTGTGGCGGTCCATGTCTACTCT | AGAGTAGACATGGACCGCCACAGGAGCCGGTTG |  |
| HA tagging | TgE1_Ap_-(C-term) | TACTTCCAATCCAATTTAATGTCTGCCTCTGCTGTTGGGCGCGAG | TCCTCCACTTCCAATTTTAGCGTTCTTGGTTTCTCCCTCGTTCATG |  |
| GFP tagging | PfE3c_Ap_ (Leader) | CCCTCGAGATGAGTTTTATAGATGAATATGATT | CCGGTACCCCTAGGTATTTCTTCTAGTAACACACCAA | XhoI, KpnI |
| GFP tagging | PfE3w_Ap_ (C-term) | TGGGGCCCGTCGACACTAGTGAAGGTTGGGAACATATGCAAAGG | CCCGGTACCCTGCAGCTCGAGGGAAGAAAAGTTGGGGAGGGGACC | SalI, PstI |
| Protein Expression | 6xHIS | GATCGAGATCGATCTCGATC | ATCCATGGCCTTACCGCTGCTATGATGATGAT | ClaI, NcoI |
| Protein Expression | PfE2_Ap_ (Full length cDNA) | AGGGGATCCCTTAAGCCGCGGATGTTCAACATAATGAGACCAAT | AGGAAGCTTTTAACTAGTGCTAGCTTAACATTTATCATCATGATATAGA | SacII, SpeI |
| Protein Expression | PfE1L_Ap_ (Full length cDNA) | AGGGGATCCCTTAAGCCGCGGATGTACCAAGTTGTCAAGGAAT | AGGAAGCTTTTAACTAGTGCTAGCTTATAAAAAGGGCAAATTTTTGAAT | SacII, SpeI |
| Gene disruption | PF13_0182 (N-term) | GGGGACAAGTTTGTACAAAAAAGCAGGCTTAATGAATATTTTGTGCATCCATAT | GGGGACAACTTTTGTATACAAAGTTGTCCTATTTCGTGAACACCA | BP clonase |
| Gene disruption | PfE3c_Ap_ (N-term) | GGGGACAAGTTTGTACAAAAAAGTTGTCGTTTACATGACTTCTGC | GGGGACAAGTTTGTACAAAAAAGCAGGCTTAATGAGTTTTATAGATGAA | BP clonase |
| Gene disruption | PfE3w_Ap_ (N-term) | GGGGACAAGTTTGTACAAAAAAGCAGGCTTAATGAGTGACAATATAGAA | GGGGACAACTTTTGTATACAAAGTTGTTTTCTTATTTTATAAGCTC | BP clonase |
| Gene disruption | hDHFR | GGGGACAACTTTGTATACAAAAGTTGTGATCAATTTATAGAAACAA | GGGGACCACTTTGTACAAGAAAGCTGGGTACTAGATTTAATAAATATG | BP clonase |

**Figure S3**

**
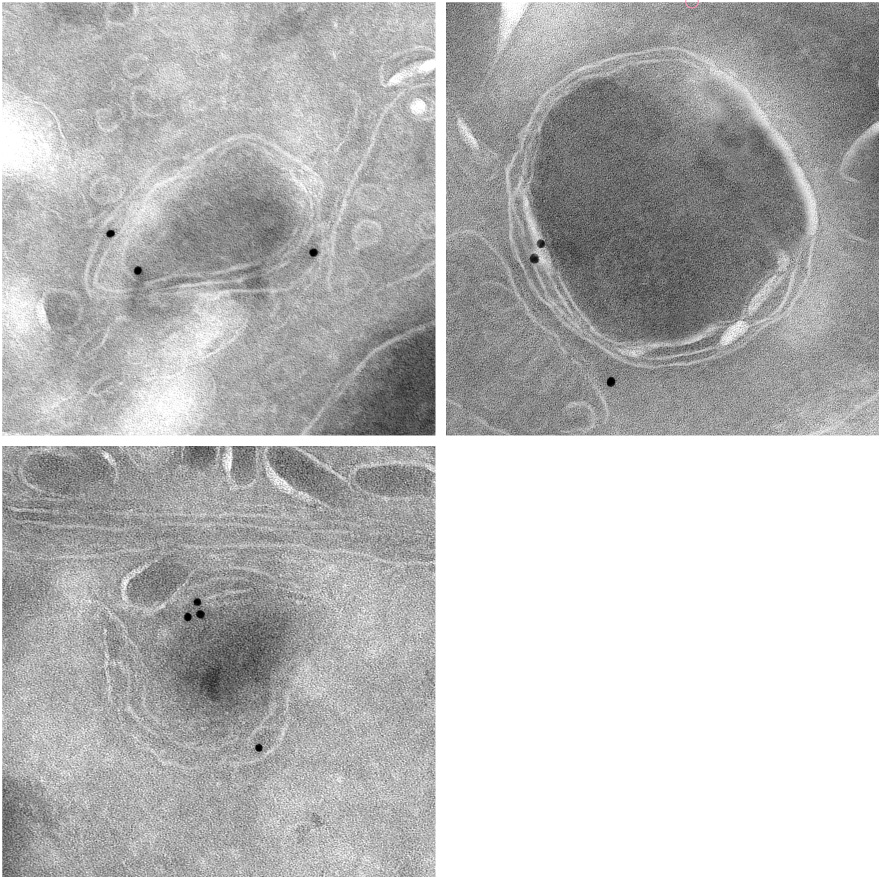
**

**Figure S4**

**Supplemental References**

1. Finn RD, Mistry J, Tate J, Coggill P, Heger A, et al. (2010) The Pfam protein families database. Nucleic Acids Res 38: D211–222. doi:10.1093/nar/gkp985.

2. Johnson LS, Eddy SR, Portugaly E (2010) Hidden Markov model speed heuristic and iterative HMM search procedure. BMC Bioinformatics 11: 431. doi:10.1186/1471-2105-11-431.

3. Sonnhammer EL, Eddy SR, Birney E, Bateman A, Durbin R (1998) Pfam: multiple sequence alignments and HMM-profiles of protein domains. Nucleic Acids Res 26: 320–322.

4. Brown NP, Leroy C, Sander C (1998) MView: a web-compatible database search or multiple alignment viewer. Bioinformatics 14: 380–381.

5. Néron B, Ménager H, Maufrais C, Joly N, Maupetit J, et al. (2009) Mobyle: a new full web bioinformatics framework. Bioinformatics 25: 3005–3011. doi:10.1093/bioinformatics/btp493.

6. Zuegge J, Ralph S, Schmuker M, McFadden GI, Schneider G (2001) Deciphering apicoplast targeting signals - feature extraction from nuclear-encoded precursors of Plasmodium falciparum apicoplast proteins. Gene 280: 19–26. doi:16/S0378-1119(01)00776-4.

7. Foth BJ, Ralph SA, Tonkin CJ, Struck NS, Fraunholz M, et al. (2003) Dissecting Apicoplast Targeting in the Malaria Parasite Plasmodium falciparum. Science 299: 705 –708. doi:10.1126/science.1078599.

8. Emanuelsson O, Nielsen H, Brunak S, von Heijne G (2000) Predicting subcellular localization of proteins based on their N-terminal amino acid sequence. J Mol Biol 300: 1005–1016. doi:10.1006/jmbi.2000.3903.

9. Nielsen H, Krogh A (1998) Prediction of signal peptides and signal anchors by a hidden Markov model. Proc Int Conf Intell Syst Mol Biol 6: 122–130.

10. Emanuelsson O, Nielsen H, von Heijne G (1999) ChloroP, a neural network-based method for predicting chloroplast transit peptides and their cleavage sites. Protein Sci 8: 978–984. doi:10.1110/ps.8.5.978.

11. Bendtsen JD, Nielsen H, von Heijne G, Brunak S (2004) Improved prediction of signal peptides: SignalP 3.0. J Mol Biol 340: 783–795. doi:10.1016/j.jmb.2004.05.028.

12. Altschul SF, Madden TL, Schäffer AA, Zhang J, Zhang Z, et al. (1997) Gapped BLAST and PSI-BLAST: a new generation of protein database search programs. Nucleic Acids Res 25: 3389–3402.

13. Edgar RC (2004) MUSCLE: multiple sequence alignment with high accuracy and high throughput. Nucleic Acids Res 32: 1792–1797. doi:10.1093/nar/gkh340.

14. Di Tommaso P, Moretti S, Xenarios I, Orobitg M, Montanyola A, et al. (2011) T-Coffee: a web server for the multiple sequence alignment of protein and RNA sequences using structural information and homology extension. Nucleic Acids Res 39: W13–17. doi:10.1093/nar/gkr245.

15. Hunter S, Apweiler R, Attwood TK, Bairoch A, Bateman A, et al. (2009) InterPro: the integrative protein signature database. Nucleic Acids Res 37: D211–215. doi:10.1093/nar/gkn785.

16. Melén K, Krogh A, von Heijne G (2003) Reliability measures for membrane protein topology prediction algorithms. J Mol Biol 327: 735–744.

17. Donald RG, Roos DS (1993) Stable molecular transformation of Toxoplasma gondii: a selectable dihydrofolate reductase-thymidylate synthase marker based on drug-resistance mutations in malaria. Proc Natl Acad Sci USA 90: 11703–11707.

18. Huynh M-H, Carruthers VB (2009) Tagging of endogenous genes in a Toxoplasma gondii strain lacking Ku80. Eukaryotic Cell 8: 530–539. doi:10.1128/EC.00358-08.

19. Sheiner L, Demerly JL, Poulsen N, Beatty WL, Lucas O, et al. (2011) A systematic screen to discover and analyze apicoplast proteins identifies a conserved and essential protein import factor. PLoS Pathog 7: e1002392. doi:10.1371/journal.ppat.1002392.

20. Agrawal S, van Dooren GG, Beatty WL, Striepen B (2009) Genetic Evidence that an Endosymbiont-derived Endoplasmic Reticulum-associated Protein Degradation (ERAD) System Functions in Import of Apicoplast Proteins. Journal of Biological Chemistry 284: 33683–33691. doi:10.1074/jbc.M109.044024.

21. Brooks CF, Johnsen H, van Dooren GG, Muthalagi M, Lin SS, et al. (2010) The Toxoplasma Apicoplast Phosphate Translocator Links Cytosolic and Apicoplast Metabolism and Is Essential for Parasite Survival. Cell Host & Microbe 7: 62–73. doi:10.1016/j.chom.2009.12.002.

22. van Dooren GG, Tomova C, Agrawal S, Humbel BM, Striepen B (2008) Toxoplasma gondii Tic20 is essential for apicoplast protein import. Proc Natl Acad Sci U S A 105: 13574–13579. doi:10.1073/pnas.0803862105.

23. Tonkin CJ, van Dooren GG, Spurck TP, Struck NS, Good RT, et al. (2004) Localization of organellar proteins in Plasmodium falciparum using a novel set of transfection vectors and a new immunofluorescence fixation method. Mol Biochem Parasitol 137: 13–21. doi:10.1016/j.molbiopara.2004.05.009.

24. Gordon JL, Beatty WL, Sibley LD (2008) A novel actin-related protein is associated with daughter cell formation in Toxoplasma gondii. Eukaryotic Cell 7: 1500–1512. doi:10.1128/EC.00064-08.

25. Vedadi M, Lew J, Artz J, Amani M, Zhao Y, et al. (2007) Genome-scale protein expression and structural biology of Plasmodium falciparum and related Apicomplexan organisms. Mol Biochem Parasitol 151: 100–110. doi:10.1016/j.molbiopara.2006.10.011.

26. Cook WJ, Jeffrey LC, Xu Y, Chau V (1993) Tertiary structures of class I ubiquitin-conjugating enzymes are highly conserved: crystal structure of yeast Ubc4. Biochemistry 32: 13809–13817.

27. Berman HM, Westbrook J, Feng Z, Gilliland G, Bhat TN, et al. (2000) The Protein Data Bank. Nucleic Acids Res 28: 235–242.
